# Computer-assisted approaches for measuring, segmenting, and analyzing functional upper extremity movement: a narrative review of the current state, limitations, and future directions

**DOI:** 10.3389/fresc.2023.1130847

**Published:** 2023-04-11

**Authors:** Kyle L. Jackson, Zoran Durić, Susannah M. Engdahl, Anthony C. Santago II, Secili DeStefano, Lynn H. Gerber

**Affiliations:** ^1^ Department of Computer Science, George Mason University, Fairfax, VA, United States; ^2^ MITRE Corporation, McLean, VA, United States; ^3^ Center for Adaptive Systems and Brain-Body Interactions, George Mason University, Fairfax, VA, United States; ^4^ Department of Bioengineering, George Mason University, Fairfax, VA, United States; ^5^ American Orthotic & Prosthetic Association, Alexandria, VA, United States; ^6^ Optimal Motion, Herndon, VA, United States; ^7^ College of Public Health, George Mason University, Fairfax, VA, United States; ^8^ Inova Health System, Falls Church, VA, United States

**Keywords:** upper extremity, functional movement, kinematic analysis, machine learning, computer vision, rehabilitation

## Abstract

The analysis of functional upper extremity (UE) movement kinematics has implications across domains such as rehabilitation and evaluating job-related skills. Using movement kinematics to quantify movement quality and skill is a promising area of research but is currently not being used widely due to issues associated with cost and the need for further methodological validation. Recent developments by computationally-oriented research communities have resulted in potentially useful methods for evaluating UE function that may make kinematic analyses easier to perform, generally more accessible, and provide more objective information about movement quality, the importance of which has been highlighted during the COVID-19 pandemic. This narrative review provides an interdisciplinary perspective on the current state of computer-assisted methods for analyzing UE kinematics with a specific focus on how to make kinematic analyses more accessible to domain experts. We find that a variety of methods exist to more easily measure and segment functional UE movement, with a subset of those methods being validated for specific applications. Future directions include developing more robust methods for measurement and segmentation, validating these methods in conjunction with proposed kinematic outcome measures, and studying how to integrate kinematic analyses into domain expert workflows in a way that improves outcomes.

## 1. Introduction

Functional upper extremity (UE) movements, where the UE is defined as including all regions distal from and including the shoulder (1), are used to purposely engage with one’s environment (2) for needed or desired activities. The execution of these movements requires the coordination of multiple processes (3), where a disruption in one part of the chain may challenge an individual’s ability to execute their desired task. Outcome measures derived from UE functional assessments (UEFAs) are used to support evidence-based research (e.g., meta-analyses), evaluate the impact of a disease or disability, and evaluate interventions (4).

The World Health Organization (WHO) International Classification of Functioning, Disability, and Health (ICF) is an internationally recognized framework for describing and measuring human health and disability (5). Using the WHO ICF, UEFAs can be classified as measuring body functions and structure (i.e., physiological function and anatomy), activity (i.e., execution of task or action by individual), or participation (i.e., involvement in a life situation), with overlap across categories being possible (4). This schema provides a standard nomenclature by which one selects outcomes measures that can link various domains (e.g., impairment, function, societal integration) to better predict relationships needed to produce desired clinical outcomes. Additionally, the ICF provides a conceptual framework for assessing function likely to be valued by the individual with a specific diagnosis or impairment. Through its direct approach for evaluating both anatomically based outcomes and their utility to a person in their environment, one can systematically assess how an intervention impacts people.

Outcome measures can be further categorized as subjective or objective. The former consists of self-reports and the latter consists of data collected by instruments or a third party using “… validated equipment and standardized measurement protocols.” (4). Both are essential for evaluating the effects of treatments. The ICF was motivated, among other factors, by the need to go beyond indicating whether a disease or disorder is present in an individual, which alone is a poor indicator of health planning and management requirements (6). In fact, the ICF was developed to augment patient evaluation and treatment from the perspective of health and not disease and disability. In other words, this approach permits a systematic documentation of an individual’s deficits and abilities. The ICF promotes a view of health that hopefully will influence policy and practice that is additive to traditional mortality and morbidity outcome measures (6).

Numerous UEFAs have been validated to provide additional information besides the presence of a disease or disorder (4, 7). However, currently validated UEFAs that measure an individual’s ability to execute a task have limitations. Although both performance-based measures and self-reports are critical, subjective self-report measures can be biased (7, 8). Furthermore, existing measures do not adequately measure movement quality (9), efficiency, or level of effort. These aspects of functional movement are important for a variety of applications, such as discerning between behavioral restitution and compensation during stroke rehabilitation (9), skilled job-related movements (10, 11), and evaluating UE prostheses (12–15).

UEFAs that use kinematics may provide more objective information of functional movement compared to existing validated clinical measures (8, 9, 16). The kinematics of human motion refer to the position displacement and its derivatives (e.g., velocity, acceleration, jerk) of the human body or manipulated objects. The analysis of kinematics includes the calculation of joint angles (17–21) and measures of functional ability during goal-oriented tasks (8, 9, 16). Kinematics have traditionally been measured using specialized equipment, such as optical motion capture systems (20–22), electrogoniometers (23), inertial measurement units (17, 24, 25), and hand-held devices (11, 26). However, many of these systems can be prohibitively expensive to own and operate or are not easily portable, restricting wide-spread usage in relevant environments. Furthermore, post-processing these information (e.g., labelling occluded markers, movement segmentation) for analysis can be a manually-intensive and time-consuming process.

Advances in measurement sensors, computer vision, and machine learning have enabled the measurement and analysis of UE kinematics beyond the laboratory. Methods have been developed for estimating human pose without markers (27–30) and automatically recognizing activities and actions (31–37). Nonetheless, there is limited development and usage of computational tools for analyzing functional UE movement kinematics that meet the requirements of domain experts (e.g., biomechanists and clinicians). For example, in 2019 the Stroke Recovery and Rehabilitation Roundtable concluded that, “… only high-speed and high-resolution digital optoelectronic systems should be used to measure kinematics…”, specifically noting that wireless wearables (e.g., IMUs), Kinect, and other optical systems are currently inadequate for measuring movement quality (9). Furthermore, validating these computational tools for use in clinical and biomedical laboratories may require a level of rigor not typical of computational fields (e.g., correlating outputs from computational tools with health-related outcomes and evaluating quantities important to movement scientists) (38, 39).

**Figure 1:**
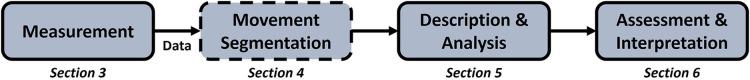
The process of analyzing functional human movement, modified from (23), which is the organizing framework for this review. Sections in this paper corresponding to the different components of the framework are indicated. The movement segmentation component has a dashed outline to indicate that it is not a necessary part of the kinematic analysis process, although it is frequently required. Definitions of components in Table 1.

This paper investigates the following question: *Given the need to inform clinical practice and job-related training with more objective data, what computer-assisted methods can reduce the burden associated with the kinematic analysis of UE movement* (see Figure 1)? Due to the expansiveness of the kinematic analysis workflow, our discussion is restricted to a few notable examples of computer-assisted approaches used in kinematic analyses as opposed to a systematic review. This paper represents an interdisciplinary perspective on the current state of computer-assisted methods as it relates to the process of conducting kinematic analyses of functional movement. Advancements needed for wider usage of kinematics for UEFAs discussed in this paper include:

1. Developing measurement approaches, such as those based on markerless pose estimation, that meet accuracy requirements of domain experts, are easy to use, and measure relevant quantities (see Section 3).
2. Computing useful measures from kinematic data often requires segmentation of movement into a standardized hierarchy, which is currently labor-intensive and not consistently defined (see Section 4).
3. The need for validated kinematics-based outcome measures (see Section 5).
4. Integrating kinematics analysis into domain expert workflows in a way that meaningfully improves domain-specific outcomes (see Section 6)

To our knowledge, a review has not been performed on computer-assisted methods for the entire UE functional movement kinematic analysis process (see Figure 1). Previous reviews have comprehensively assessed kinematic measures that quantify UE performance during a variety of functional tasks (8, 16, 40), although these do not consider the end-to-end kinematic analysis workflow. Related reviews cover multiple components of the kinematic analysis workflow (33, 40, 41), but they either are focused on a specific application (e.g., handwriting (42)) or omit important components of the workflow (e.g., functional primitive segmentation (40) and kinematic measurement (41)). There have also been reviews of computer-assisted methods to support rehabilitative training using serious games (43, 44), which is related to our review but is not the focus.

## 2. Review organization

### 2.1. Exclusion and inclusion criteria

Excluded are applications in sports (45) and hand gesture recognition (46, 47). Hand gesture recognition is excluded because it is a form of non-verbal communication, as opposed to being used for assessing functional UE movement.

Job-related assessments of skillful UE functional motion using kinematics are included. These assessments are similarly motivated by the need for more objective measures of performance (10, 11) and follows closely with the health-oriented kinematic analysis workflow. The methods developed for job-related assessment applications can also be applied to health applications involving the UE.

### 2.2. Organizational overview

Winter (23) describes the scientific approach to biomechanics, which this paper uses to represent the kinematic analysis workflow associated with UEFAs (23). We make an addition to the kinematic analysis process to include movement segmentation, which has previously been identified as necessary for a variety of kinematic analyses (40, 48). The resulting process (see Figure 1) consists of movement measurement (Section 3), segmentation (Section 4), description and analysis (Section 5), and assessment and interpretation (Section 6). Definitions for each component are in Table 1.

**Table 1:** Computer-assisted functional upper extremity assessment process modified from (23).

Process phase	Definition
Measurement	The capture of UE motion kinematics, which results in data used for analysis. Often done with optical motion capture systems, wearable inertial measurement units, commodity cameras, or taken directly from the object being manipulated (e.g., a tablet stylus pen).
Movement Segmentation	The process of segmenting movements into distinct movement phases, such as functional movements and primitives (see Table 3).
Description	Can be of many forms, but typically defined as visualizations of the data (e.g. velocity magnitude time series of wrist marker) or simple outcome measures.
Analysis	Defined as a mathematical operation performed on the data to present them in a different form or to combine several sources of data to produce a variable that is not directly measurable (e.g., inverse kinematic solution).
Assessment and Interpretation	The assessment of descriptions and analyses, which informs decisions about interventions.

## 3. Measurement

### 3.1. Background

Kinematics is concerned with quantifying the details of movement itself (e.g., position displacement, velocity, acceleration, and jerk) and not the forces that cause the movement, where the goal is to use kinematics to provide actionable information for the domain expert. Kinematic data are collected by either direct measurement or optical systems (23).

#### 3.1.1. Direct measurement systems

Direct measurement systems involve placing equipment on the individual being evaluated, which includes using electrogoniometers and special gloves for hands outfitted with transducers for measuring joint angles, and inertial sensors (23). These direct joint angle measurements (17, 18) can be used in a variety of ways (e.g., visualized or measures computed from joint angle time series) to evaluate functional movement (19). Electrogoniometers can be relatively inexpensive and provide kinematic data immediately. However, it can be challenging to properly place the goniometer on an individual and wearing the device can influence their natural movement due to encumbrance. Additionally, more complex goniometers may be required for joints that do not move as hinge joints (e.g., wrist and shoulder). Inertial sensors are worn on the body, where inertial data from the sensors individually (e.g., motion of the wrist only) (49) or from multiple sensors (e.g., fused together to provide human pose estimates and joint angles) can be used for kinematic analyses (25, 50). There are also systems that measure the movement of a device being operated by the individual, such as end effectors (e.g., tablet pens (42), haptic devices (26), ultrasound probes (11), laparsoscopic manipulators (51, 52)) and exoskeletons (53)). The equipment cost and ease of use varies greatly across these systems, but generally they provide high sampling rates and accurate kinematics.

#### 3.1.2. Optical systems

**Figure 2:**
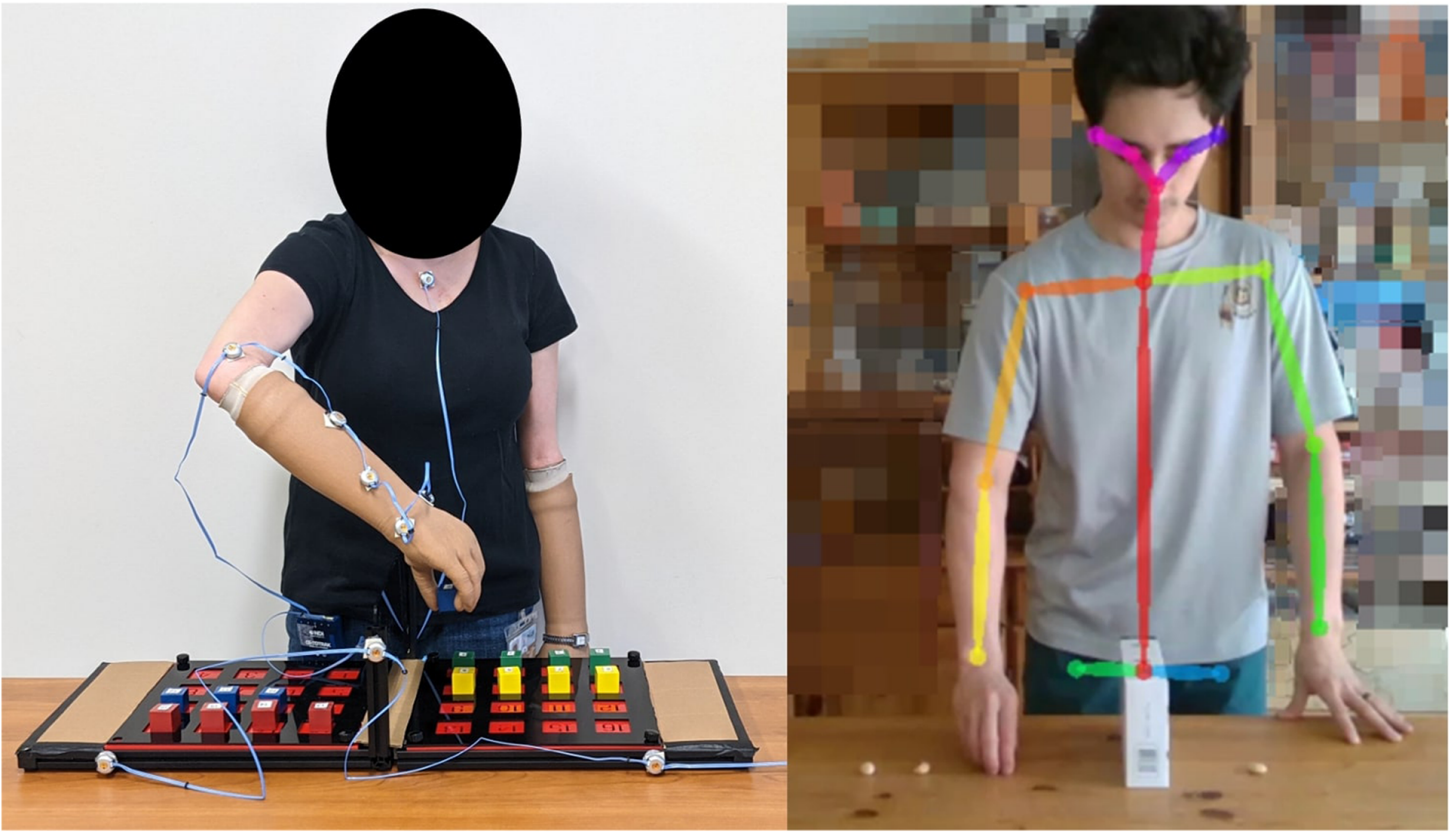
(left) Individual outfitted with active markers for an optoelectronic motion capture system (NDI Optotrak®) while completing the Targeted Box and Blocks Test (54). (right) Individual moving objects over the middle partition while being tracked with the markerless pose estimation tool OpenPose (28). In the left image, multiple markers are placed on the right arm to reduce tracking fragmentation due to occlusions. These markers, cables, and associated outfit requirements could encumber or impact the individual’s normal movement, motivating the use of a markerless motion capture system. Participant consent was given for photo usage.

Optical systems can be categorized as being markerless video capture, marker-based capture with passive reflective markers, or optoelectric systems with markers that actively emit light (23). Optical systems are used to provide motion of individual landmarks (e.g., on the wrist) or to model human pose, where the latter can be used to measure joint angles (19). Markerless capture cameras, which include 2D RGB and 3D RGB-D cameras, are relatively inexpensive, but have traditionally required anatomical landmarks to be manually identified by a human operator, an approach that makes this process infeasible for large studies or widespread usage. However, markerless and passive marker systems either do not or minimally encumber the individual being evaluated, whereas active markers can be encumbering due to the wiring between the markers (see Figure 2). Multi-camera systems for passive and active markers can also be prohibitively expensive to own and operate, although these systems are highly accurate and are considered to be the “gold standard” in movement science (9, 55). Although not an optical system, some systems use active markers that emit sound or radio signals, which are picked up by receivers used to locate the active marker (13).

### 3.2. Human pose estimation

Measurement tools are needed that minimize the impact of encumbrance on natural movement, provide near real-time kinematic data with minimal noise and inaccuracies, and are relatively inexpensive to own and operate. A 2019 systematic review of low-cost optical motion capture for clinical rehabilitation indicated the need for better measurement methods and validation studies, although most papers reviewed were not specific to UE functional motion (56).

There has recently been substantial progress on 2D and 3D human pose estimation using low-cost sensors, where the goal is to infer a representation of the body from images, video, or inertial sensor data. Kinematics can then be derived from the output representation. Table 2 represents a taxonomy of these methods, inspired by previous taxonomies (30, 57). Included in Table 2 are recent reviews and research papers for each methodological approach, along with a non-exhaustive list of works evaluating the utility of these methods for measuring UE kinematics. A comprehensive review of human pose estimation algorithms is beyond the scope of this paper, where instead we include a brief description of recent measurement approaches categorized by one of three input data types—RGB, RGB-D, and inertial data—and synthesize recent results from studies evaluating their utility for use cases involving UE functional motion. Radio frequency devices (e.g., WiFi) that do not require transmitters placed on the body have also been used for pose estimation (30). However, these methods currently have low spatial resolution, and we are not aware of their usage for UEFAs.

**Table 2:** Taxonomy of human pose estimation approaches inspired by (30, 57).

Output dimension	Output representation	Measurement device	Input data	Methodological references	UE application
2D HPE	Planar	Monocular camera	2D RGB	(30, 57)	Not aware of usage
	Keypoint	Monocular camera	2D RGB	(28, 30, 57)	(17, 58, 59)
3D HPE	Kinematic	Monocular camera	2D RGB	(30); (60) for hand pose	(59)
		Multi-view cameras	2D RGB	(30, 61, 62)	(63, 64)
		Depth camera (e.g., Kinect)	3D RGB-D	(30); (60) for hand pose	(59, 65–71, 83, 84)
		Inertial sensors	Inertial data	(25, 50, 72)	(17, 24, 73, 74)
	Volumetric	Monocular camera	2D RGB	(29, 30, 75, 76); (60) for hand pose	(17)
		Multi-view cameras	2D RGB	(30)	Not aware of usage
		Depth cameras	3D RGB-D	(30, 77)	(78)

HPE, human pose estimation; RGB, red-green-blue; RGB-D, red-green-blue-depth; 2D, two-dimensional; 3D, three-dimensional.

Additionally, we consider four representations used in human pose estimation—planar, kinematic, keypoint, and volumetric—along with their respective input data types (30) (see Table 2 for associations between representations and input data):

∙ **Planar**: This representation models the shape and appearance of the human body, which is usually represented as rectangles approximating the contours of the body.
∙ **Kinematic**: Models the joint positions and limb orientations of the human body in a 3D graph representation.
∙ **Keypoint**: Similar to the 3D kinematic representation, except that it is a 2D projection of the 3D body (see Figure 2), i.e., the inferred representation is only in 2D. Note that some works in the computer vision literature (30) conflate the 3D kinematic representation with the 2D keypoint representation, which can be confusing.
∙ **Volumetric**: A 3D mesh representation.

#### 3.2.1. 2D RGB input

Human pose estimation algorithms can take input two-dimensional (2D; x and y) red-green-blue (RGB) images, which is what most consumer cameras capture, and output either a 2D or 3D representation of the body (30). Per Table 2, the output 2D representations are either planar or keypoints, and output 3D representations are kinematic or volumetric. Large data sets consisting of labeled anatomical landmarks or human pose are used to train machine learning models that infer anatomical landmarks in new, unseen images.

##### 3.2.1.1. 2D keypoint representation

The output 2D keypoint representation has had considerable research interest recently, which is partially motivated by the ubiquity of RGB cameras (30, 57). Although there are many algorithms, one notable 2D human pose estimation algorithm is OpenPose (28), which has been evaluated for utility in measuring UE kinematics (17, 58, 59), among other approaches. These applications involved evaluating the 2D errors of the pose predictions for reaching movements in infants (58) or extracting depth values from a red-green-blue-depth (RGB-D) image using the 2D predictions to create 3D landmarks of UE movements (17, 59). Using 2D keypoint predictions followed by converting to 3D coordinates using depth from an RGB-D camera appears to be the most common use of 2D pose estimation by movement scientists because human functional motion is often tri-planar, except for assessments where uni-planar movement is specifically of interest (e.g., shoulder abduction in frontal plane (59)).

The best-performing 2D pose estimation algorithms have been demonstrated to be useful for a variety of training and rehabilitation applications (17, 58, 59, 79, 80) involving gross movements. However, improvements are still needed to make 2D pose estimation comparable to gold standard motion capture systems, such as incorporating physiological constraints (17, 80) and temporal smoothing (17). Additionally, many of the pre-trained 2D pose estimation methods rely on training data sets consisting primarily of able-bodied individuals (17) with crowd-sourced, hand-labeled keypoints that potentially contain errors (38). Perhaps the greatest limitation of 2D pose estimation for analyzing human kinematics is that it is not 3D, which makes measurement of complex 3D motions (i.e., tri-planar), textures, and shapes infeasible, particularly when using a single RGB camera.

##### 3.2.1.2. 3D kinematic representation

An output 3D kinematic representation can be inferred from 2D RGB images either directly or as a follow-on step to an intermediary 2D pose estimation output (i.e., “lifting” from 2D-to-3D), where 2D-to-3D lifting approaches typically outperform direct estimation methods given the current state-of-the-art 2D pose estimation methods (30). Alternatively, 2D pose estimation using RGB from multiple camera views of the individual can provide an estimate of a 3D kinematic representation (61–64), where a multi-camera setup requires synchronizing the recordings and computing 3D keypoints from the triangulation of the synchronized 2D keypoints (62). Additionally, multi-camera setups minimize the possibility of body parts being occluded during more complex motions, where occlusions can cause instability in pose estimation performance (62, 78). These multi-camera methods have been evaluated against marker-based optical motion capture for the UE (61–64). Although they were not focused solely on UE movement, the assessment methodologies and results are relevant.

OpenPose (28), a popular 2D pose estimation method from RGB, was evaluated with a multi-camera setup during walking, jumping, and throwing a ball, where the tracking results were compared to a marker-based optical motion capture system (63). For the shoulder, elbow, and wrist joints tracked, (63) found the respective mean absolute error (MAE)—where we calculated the mean and standard deviation of the reported MAE values across activities and axes from Table 1 of (63)—means to be 23.2, 28.9, and 24 mm, and standard deviations to be 9.29, 16.2, and 13.5 mm. In a separate study, three 2D pose estimation algorithms in a multi-camera setup outputting 3D pose estimates during walking, running, and jumping were compared to marker-based optical motion capture (64). The minimum and maximum of the 95% limit of agreement values reported for the shoulder joint center during walking were 14 and 43 mm, respectively, with generally higher errors for running and jumping. Ivorra et al. (59) evaluated the application of multiple pose estimation approaches to tracking UE exercises with a single camera view and found the method that used only 2D RGB data (referred to as RGB-3DHP)—percent difference averaged across tasks of 18.2% compared to marker-based capture—to be less accurate than the other methods—10.7% and 7.6%—that used RGB-D data as input. Therefore, a 3D kinematic representation output from 2D images may be currently restricted to measuring gross UE motions for applications where high accuracy is not required, such as rehabilitation games, as recommended by (59).

Regarding the value of estimating 3D pose from multiple RGB cameras, (62) found that for a 2D pose estimation method called HRNet, the average marker error across all markers—where markers were for the whole body—and activities was 32mm with the two-camera setup, and improved to 29 mm with a five camera setup. However, accuracy was consistent across the varied pose detectors and number of cameras when using OpenCap (proposed by (62)), which makes some modifications to the pose estimation process. These results suggest that while multiple cameras will help resolve issues with occlusion, exactly how many cameras are needed will depend on the pose estimation method being used and the types of motions being measured.

##### 3.2.1.3. 3D volumetric representation

Inferred 3D volumetric representations (29, 75, 76) from 2D RGB input appear to be not as thoroughly studied for measuring UE kinematics compared to the 2D keypoint and 3D kinematic representations, although these methods appear to capture details of hands relatively well. One UE application example is UE kinematics being measured with wearable IMUs using an inferred 3D mesh representation and 2D keypoint representation—stereo was used to get the depth values for the 2D representation—for IMU calibration (17). After calibration, the IMUs could be used to track joint trajectories alone or optionally with the 3D pose estimates from video.

#### 3.2.2. 3D RGB-D input

Pose estimation methods that take input 3D red-green-blue-depth (RGB-D; x, y, and z) data will output either 3D kinematic or volumetric representations of the body (see Table 2). Microsoft Kinect—versions include V1, V2, and Azure—is an RGB-D camera commonly used in studies evaluating pose estimation for kinematic measurements because the cameras are relatively cheap, portable, easy-to-use, and have a built-in pose estimation capability that returns inferred joint positions using depth data. Details about the Kinect V2 pose estimation algorithm are published (81), whereas the details for the Azure Kinect are not disclosed. Different versions of the Kinect are used in all reviewed papers using RGB-D to measure UE kinematics (59, 65, 67, 69, 70, 78) except for (17) which computes the depth map from calibrated stereo cameras. Compared to pose estimation methods that use 2D RGB images only, methods that use RGB-D images appear to provide more accurate estimates (59) when only a single camera is used, which accurate estimates are necessary for measuring fine movements. However, this may not be the case in multi-camera setups and is a subject for further study.

##### 3.2.2.1. 3D kinematic representation

For the output 3D kinematic representation, none of the UE application studies we review in this category (59, 65–71, 82–84) involved other pose estimation algorithms that use depth to infer body pose, although algorithms exist (30). The consensus from these studies, which involved a variety of UE movements, is that the Kinect’s pose estimation method is suitable for measuring gross movements but is not suitable for fine movements. For example, the Kinect failed to adequately track shoulder movement (69), which is an important compensatory movement to measure in clinical settings (e.g., during stroke rehabilitation (78) and UE prosthesis use (12–14)). Better methods could be used if real-time processing is not a requirement (59), whereas the Kinect was developed specifically for gaming and therefore requires real-time pose estimates.

##### 3.2.2.2. 3D volumetric representation

3D volumetric representations can also be inferred or fitted from RGB-D images (30, 77, 78). Jatesiktat et al. (78) proposed improving the Kinect V2 3D kinematic pose estimates for the upper body by fitting a human mesh representation (85) to the depth image, along with using two wrist-worn IMUs to mitigate issues with forearm occlusion. This approach allowed for better tracking of the shoulder, wrist, and elbow compared to using the Kinect pose tracker alone by 25.9% across all the evaluation data and 43.7% across the cases with occlusion. While (69) indicated the Kinect pose tracker alone could not provide measurements of fine shoulder movement, the results from (78) suggest that a 3D volumetric representation can be used to improve 3D kinematic representations. According to Figure 4 in (78), the proposed method with the IMU improved the average error from approximately 45 to 33 mm, although whether this is sufficient for fine shoulder motion is an open question.

#### 3.2.3. Inertial data input

Wearable IMUs have been studied extensively by the movement science community (17, 24, 25, 49, 50, 72–74, 78, 86). Although kinematics from the IMUs in isolation can be used (e.g., motion of the wrist-worn IMU), multiple IMUs attached to the body are used for pose estimation due to sensors being low cost and not suffering from issues associated with occlusion. IMUs have been assessed to be suitable for estimating UE kinematics in the laboratory and clinical settings (24, 50, 74), but there are challenges associated with widespread usage outside of these controlled settings.

These challenges include sensor calibration, drift over time associated with gyroscopes, and magnetometers being sensitive to certain metals in the environment (25, 50, 72). However, a variety of methods exist for calibration, reducing drift, and handling magnetic disturbances (25), where the extent of these issues (e.g., magnitude of the drift) will depend on what methods are used. For example, (73) excluded magnetometers from their proposed upper body pose estimation method using IMUs, avoiding magnetometer disturbance concerns, although a comparison of the magnetometer-free method with methods using magnetometers while attempting to minimize magnetic disturbances (25) was not performed. Newer methods that fuse optical motion capture with IMUs for calibration could make it easier to get relevant kinematic measurements of UE movement (17). Inertial data, potentially along with other data types that could come from wearables (e.g., electromyography), can also be fused with optical pose estimation approaches to provide potentially better kinematic measurements (17, 30).

### 3.3. Measurements of manipulated systems

While using optical motion capture and IMUs to measure complex functional UE movement kinematics are popular, there are other ways to measure functional UE movement that tend to be application-specific. For example, haptic virtual environments record precise kinematic information via encoders (87) and provide a customizable workspace to assess functional UE movement, which has uses in rehabilitation (26) and surgical skill assessment (88). Kinematic measurements have also been recorded from real laparoscopic box trainers, which has been used to evaluate surgical skill (51, 52, 89–93). UE kinematic measurements have been recorded by the objects people manipulate, as is the case with ultrasound probes that have been used to assess the skill of obstetric sonographers (11). Handwriting on digitizing tablets are used for assessing neurodegenerative diseases (42, 94) and dysgraphia (95) using kinematic information of the pen tip and pen pressure on the writing surface.

### 3.4. Evaluating measurement methods

Domain experts need to understand how well measurement systems work and whether they can be adopted for their applications. The computationally-oriented literature tends to focus on evaluating the accuracy and run-time of new measurement methods, such as assessing keypoint localization error for 2D pose estimation using the keypoint representation (30). For adoption in healthcare applications involving UEFAs, test-retest reliability (50, 68, 70, 96) and validity (24, 50, 59, 66, 68) need to be assessed. Furthermore, accuracy assessments (25, 63, 64, 67, 73, 74, 78) that do not rely solely on healthy participants (17, 38) are needed. Although the reviewed pose estimation methods are finding utility in health applications related to measuring UE movement, more widespread adoption of these tools require further assessments of measurement accuracy, validity, and reliability (9) on quantities that are important to movement scientists (38), e.g., joint angle (17–21, 50, 62).

## 4. Movement segmentation

### 4.1. Background and motivation

#### 4.1.1. Background

Useful kinematic descriptions and analyses require comparing the same types of functional motions across individuals, such as the reaching portion of a trajectory an individual follows to grasp an item (97). However, movement segmentation is challenging because the UE is complex (e.g., the UE has seven degrees of freedom and can be moved with the torso) and people move differently, even on the same task (48). Due to the variability in UE movement on a given functional task, segmenting the movement into meaningful parts for analysis is a manually intensive process and can be the most time-consuming part of the kinematic analysis process. Therefore, targeting research efforts to alleviate the burden of segmenting kinematic data would have considerable impact on the kinematic analysis process across many applications.

A segmentation procedure has two outputs: (1) the start and stop timestamps of the motion sequence and (2) what type of motion the sequence is (98–100). This requirement means that a sequence can potentially have multiple classes of motions, which is considered a more challenging problem than predicting the motion class of an already trimmed segment consisting of only one class. The simplest and most time-consuming way to do this is to manually segment the data based on descriptions of the movement (see “Describe” in Figure 1) and video recordings (48). The development and usage of automated movement segmentation algorithms will help reduce the cost and burden associated with conducting kinematic assessments, especially as kinematic assessments become more prevalent and the amount of data needing processing exceeds the capacity of current workflows used in the research setting.

#### 4.1.2. Organizing hierarchy

The computational literature inconsistently labels levels of functional motion, which can be confusing when trying to identify which segmentation methods are suitable for a particular application. For example, the definition of an action in (34) differs from (101), where (101) analyzes more complex activities. A recent partonomy-based activity recognition method proposed a general structure for categorizing human movements, which included the activity, sub-activity, and atomic action categories (102). These categories are comparable to the hierarchical levels adopted by (2) in their UE functional motion hierarchy, which includes activities, functional movements, and functional primitives. This review is targeted towards helping computational researchers better understand the kinematic analysis process for UEFAs and domain experts understand the tools that are available to them. Therefore, this review follows the terminology from the health literature and adopts the hierarchy from (2) to organize the reviewed segmentation approaches, with other researchers also recommending this hierarchy for segmentation (103).

The UE functional motion hierarchy (see Table 3) used in this paper has the following three levels: *activities*, such as eating dinner; *functional movements*, such as drinking water or tasting a spoonful of soup; and *functional primitives*, which are short and discrete movements, such as reaching, transport, grasping, stabilizing, idling, and repositioning (2). This hierarchy captures the idea that functional motions can be decomposed into different levels of motion with decreasing duration and complexity, with the more granular motions serving as building blocks for more complex motions.

**Table 3:** Upper extremity functional motion hierarchy (2).

Hierarchy layer	Goals (i.e., tasks)	Duration	Examples
Activities (broad, see Section 4.3)	Many	Minutes to hours	∙ Cooking dinner ∙ Bathing ∙ Putting clothing on
Functional movements${}^{a}$ (see Section 4.4)	Few	Seconds	∙ Tasting sauce ∙ Putting arm through sleeve ∙ Zipping up jacket ∙ Tying shoelace
Functional primitives (granular, see Section 4.5)	One	Sub-seconds to seconds	∙ Reach ∙ Reposition ∙ Grasp ∙ Transport ∙ Stabilize ∙ Idle

${}^{a}$Has also been referred to as actions (2).

#### 4.1.3. Necessity of different segmentation levels

**Figure 3:**
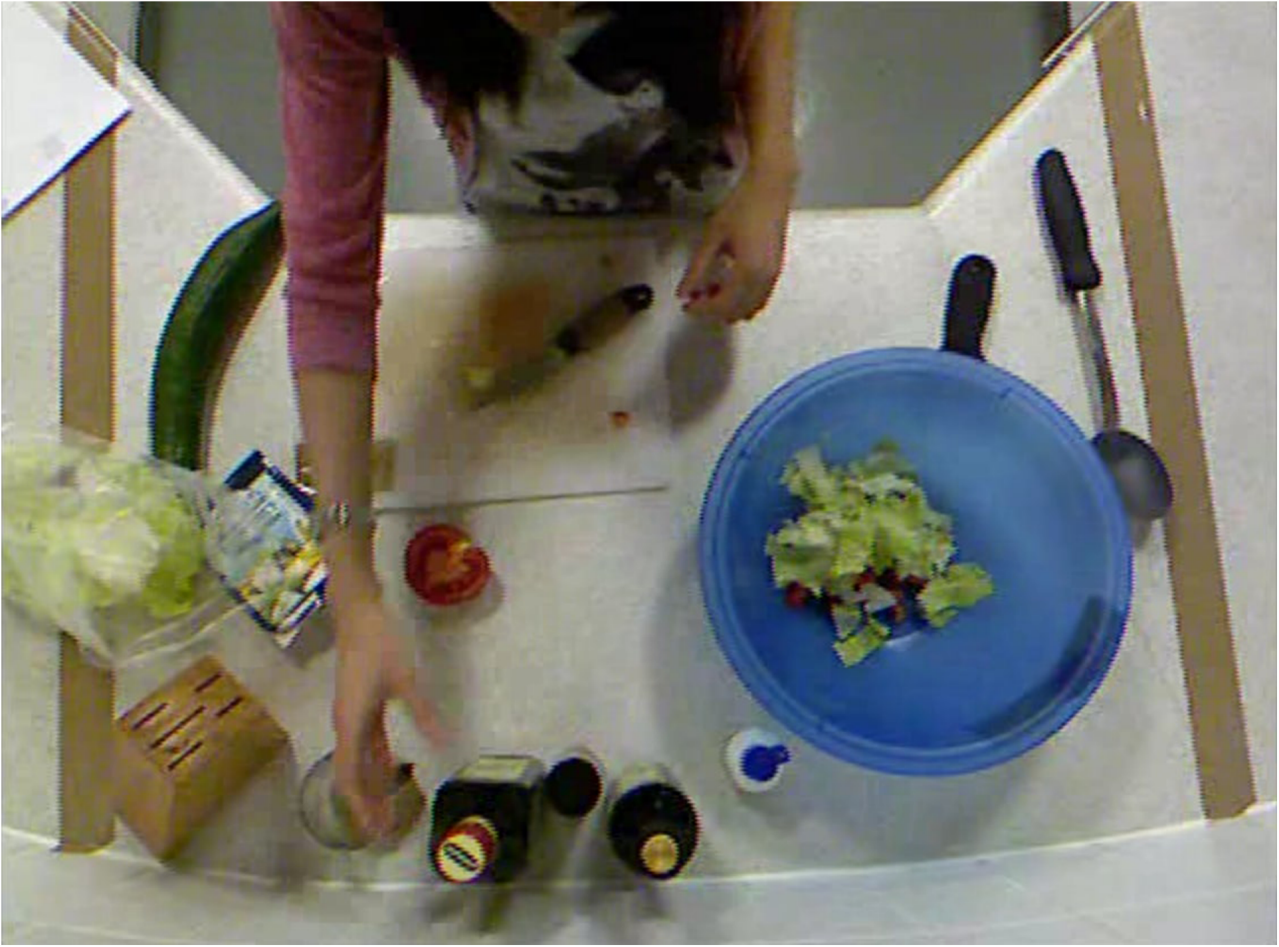
Single frame from the 50 Salads data set (104) (license under a Creative Commons Attribution-NonCommercial-ShareAlike 4.0 International License: https://creativecommons.org/licenses/by-nc-sa/4.0/), which required individuals to make salads. Data includes RGB-D video, accelerometer data from utensils, and functional movement labels.

Suppose a rehabilitation specialist is interested in evaluating the kinematics associated with how individuals make a salad within a standardized setup (see Figure 3). The activity is known—making a salad—but there are multiple tasks an individual must do, such as grabbing a bottle of vinegar for the dressing and cutting a tomato. Ideally, these different tasks would be segmented so that kinematic measures can be used for comparison for the same task, either across groups or over time. One option is for the clinician to do this manually, but that is time consuming. Another option is to use algorithms that automatically identify these different tasks. These algorithms address the problem of **functional movement segmentation**.

Suppose the tasks have now been segmented and the clinician would like to analyze the functional primitive kinematics of how individuals reach for and grasp the bottle of vinegar (e.g., some kinematic analyses require primitive segmentation (97, 105, 106)). This reaching motion consists of multiple functional primitives (see Table 3) and would need to be segmented. Again, one option would be to manually segment the primitives (48). However, there are algorithms that focus specifically on automating **functional primitive segmentation** (e.g., (26, 106)). Given the distinction between the algorithms for segmenting functional movements and primitives, one section is dedicated to each in this review. Furthermore, functional movement segmentation algorithms are usually disjoint from functional primitive segmentation algorithms.

##### 4.1.3.1. An exception

Although movement segmentation is widely done in the health and computational literature, e.g., some measures of movement smoothness require it (105), there are some examples of computational approaches that skip movement segmentation altogether (51, 52, 107). Kinematic measures of surgical skill from the entirety of each surgical training task have been used (51, 52), i.e., motions during tasks were not segmented. Similarly, (107) proposed a surgical skill evaluation approach that explicitly does not require segmentation.

### 4.2. Data sets

Table 4 includes publicly available data sets with labeled activity and functional UE movements, although most of these data sets also include non-UE motion. These data sets have sequences with potentially multiple segment classes, requiring temporal segmentation. We are not aware of publicly available data sets with labeled motions found in clinically validated UEFAs, which are necessary for reducing the burden associated with the UE kinematic analysis workflow. Lin (108) identified rehabilitation-focused data sets for the UE and lower extremity. However, the only rehabilitation-focused UE functional motion data set (109) could not be found online. Zhang et al. (99) and Hu et al. (100) include state-of-the-art action detection performance measures for a variety of data sets, and most of the referenced data sets in Table 4 include benchmark performance results using supervised and unsupervised approaches.

**Table 4:** Publicly available activity and functional motion data sets with segment labels.

Data set	Topic	Task(s)	Participants	System	Labels
JIGSAWS (91)	Surgical activity	Suturing, knot-tying, needle passing	8 surgeons of varying skill	Robotic kinematics; stereo video	Surgical skill; functional movements and primitives
50 Salads (104)	ADL${}^{a}$	Food (salad) preparation	27 able-bodied individuals	Accelerometry from objects; RGB-D	Functional movements
Breakfast Actions (110)	ADL	Varying cooking tasks	52 able-bodied individuals	Markerless video capture	Activities and functional movements
EGTEA Gaze+ (111)	ADL	Varying cooking tasks	32 able-bodied individuals	Markerless video capture with gaze tracking	Functional movements
TUM Kitchen (112)	ADL	Object interaction	4 able-bodied individuals	Markerless video capture; RFID on objects	Functional movements
UW IOM (113)	ADL	Object interaction	20 able-bodied individuals	Kinect RGB-D camera	Functional movements
LARa (114)	Logistics	Picking and packaging	14 able-bodied individuals	Maker and markerless video capture; IMUs	Activities and functional movements
CAARL (115)	Logistics	Picking and packaging	2 able-bodied individuals	Marker and markerless video capture on person and objects	Activities and functional movements
AVA-Kinetics (116)	Varied	Varied object and person interactions	Not reported (large data set)	Markerless video capture (Youtube)	Activities and functional movements
Something-Something V2 (117, 118)	Varied	Object interaction	Not reported (large data set)	Markerless video capture (crowd sourced)	Activities and functional movements
HMDB51 (119)	Varied	Varied object and person interactions	Not reported (large data set)	Markerless video capture (Youtube, movies)	Activities and functional movements
UCF101 (120)	Varied	Object and human interaction; body motions	Not reported (large data set)	Markerless video capture (Youtube)	Activities and functional movements
MOMA (102)	Varied	Object and human interaction	Not reported (large data set)	Markerless video capture (Youtube)	Activities and functional movements
Action Genome (121)	Varied	Object interaction	Not reported (large data set)	Markerless video capture (Amazon Mechanical Turk)	Activities and functional movements
Ego4D (122)	Varied	Varied, including person and object interaction	923 participants from multiple countries	Egocentric RGB, IMUs, gaze, and audio	Activities and functional movements
BEHAVIOR-1K (123)	ADL	Object interaction	None (simulation)	Simulation	Activities

${}^{a}$Activities of daily living (ADL).

### 4.3. Activity segmentation

Activities are the highest level in the functional motion hierarchy (see Table 3). Activity recognition (35, 36, 102) is useful for assessments where individuals are being evaluated in their natural environments throughout the day, where it may be useful to automatically identify activities an individual is doing. At-home health monitoring is especially important to clinicians because improvement in clinical measures does not necessarily mean UE performance improvement in free-living and unstructured environments (49), where improvements in the latter is the goal.

Human activity recognition has received attention from the computational research community; however, many of the methods need labeled data, which are not rehabilitation specific (see Table 4). Additionally, activity recognition as part of UEFAs is a relatively undeveloped area. Inaccurate commodity measurement systems (e.g., wearable sensors), non-validated outcome measures, and human factors challenges are current barriers to use of data capture and analysis (86). Furthermore, current measures used for at-home UEFAs are not activity-specific and instead summarize different aspects of UE usage throughout the day (49, 103). Activity recognition methods are useful for at-home UEFAs (84, 103), but their utility is not as well defined compared to the automated segmentation of functional movements and primitives (see Table 3). This paper does not thoroughly review human activity recognition methods due these aforementioned issues. Additionally, the activities performed during UEFAs, which are most commonly done in clinics, research labs, or as part of job-related assessments and training, are pre-defined to include only the activities of interest.

### 4.4. Functional movement segmentation

Functional movement segmentation approaches, also known as human action detection in the computational literature (98–100), typically use supervised or unsupervised learning. Progress in functional movement segmentation algorithms for UE functional motions has benefited from the availability of labeled data sets. Algorithmic development has therefore been largely focused on these well-annotated data sets because it is easier to compare and evaluate algorithms.

#### 4.4.1. Supervised learning

Combined segmentation and classification has been approached from a supervised learning perspective using only kinematic data (124, 125), kinematic and video data together (124, 125), or video data alone (124–126). These approaches have the goal of densely labeling all time-steps in the sequential data with functional movement class out of multiple classes. This differs from computational methods that assume the start and end points of the segments are given (127, 128), thereby reducing the problem to simply classifying the given segments. However, this is not a reasonable assumption for real-world UEFA use cases. Additionally, kinematic information alone has primarily been used in the health literature to segment movement (48), whereas contextual features related to the objects being manipulated (e.g., distance from hand to nearest object) have been used for segmenting surgical motions (125). These supervised learning algorithms may also work for a variety of functional movements if labeled data are available, as is done in (125).

#### 4.4.2. Unsupervised learning

Unsupervised approaches to movement segmentation do not require ground truth labels for training but tend to assume that there are repeated patterns in the movements (129, 130). These methods can also use a variety of data sources, such as only the end effector kinematics (131) or both kinematic and video features (129) for robotic surgery motion segmentation. More general unsupervised segmentation approaches can also use the whole body pose (i.e., multiple anatomical landmarks) (130).

### 4.5. Functional primitive segmentation

Lin et al. (48) provides an organizing framework for functional primitive segmentation, which includes online and offline methods. A variety of approaches are reviewed in (48) for general primitive segmentation (i.e., includes full-body primitives and gesture recognition) that apply to a variety of tasks (i.e., many UE functional motions require reaching, grasping, etc.). This section focuses specifically on methods for UE functional motion, either of the UE or an end effector.

Feature vector thresholds and zero-crossings (48) work well for simple actions and small data sets that allow researchers to visually verify the movement segments. Engdahl and Gates (97) segmented functional UE movement during pre-defined activities of daily living (ADLs) into reaching and object manipulation phases using a fixed-velocity magnitude threshold. Cowley et al. (12) and Engdahl and Gates (15) evaluated UE prosthesis users compared to able-bodied individuals on a set of standardized ADLs and segmented the movement primitives using pre-defined velocity magnitude thresholds. Li et al. (132) accounted for differences in participant kinematics while transporting objects by selecting 50% of movement time as when the hand reached a target position.

Approaches that use thresholds and zero-crossing tend not to perform well with complex functional movements (48), particularly for reaching motions (133). Some measurement systems allow for the collection of events (i.e., additional context about what the individual is doing, such as making contact with objects) in addition to kinematics, such as in haptic virtual environments. These events can be used to indicate action segments, e.g., person grasped object, person released object (26). Jackson et al. (26) showed that primitive segmentation, such as reaching and grasping, using a fixed-velocity magnitude threshold can result in incorrect primitive segments, requiring more robust computational approaches. To remedy this, (26) proposed a movement primitive segmentation approach that uses distance from the object and event recordings to segment reaching from object manipulation. This method has since been used to segment the reach and dwell primitives of pen point trajectories during the Trail Making Test to assess cognitive function (106).

Additional approaches to segmenting UE movement primitives include using 2D hand trajectories for identifying different hand-drawn shapes by segmenting the trajectory into strokes based on large changes in the angle between line segments and the horizontal axis (134). Motivated by robotic imitation learning, visual information, specifically kinematics derived from the Kinect pose estimation software, has been used to segment functional UE movements into reaching, manipulation, and release (135).

### 4.6. Evaluating segmentation performance

Given labeled data sets for both functional movements and primitives, segmentation evaluation measures include accuracy, precision, recall, overlap between ground truth and predicted segment classes, and the ordering of predicted segments (48, 98, 125). Unsupervised and supervised functional primitive segmentation algorithms can use the same data sets for evaluation (e.g., as has been done with JIGSAWS (91)). However, due to challenges associated with creating ground truth labels for functional primitives, verification of temporal segmentation results is limited (48). One of the major challenges with acquiring data sets of motion primitives is that it is still unclear what separates the different primitive phases using kinematics alone, especially given variations in pathologies, impairments, and movement strategies. Similarly, functional movement labels are not reliably identified across raters (98). Additionally, many of the available data sets focus on healthy, able-bodied populations, which may not properly indicate whether a segmentation approach will generalize to populations of interest to domain experts (48).

## 5. Description and analysis

The description and analysis phase (see definitions in Table 1) of the kinematic analysis process (see Figure 1) converts the measured and segmented kinematic data into a format usable by a domain expert to inform their decisions about treatment or interventions. The measurement accuracy and segmentation requirements for specific descriptions and analyses informs what measurement and segmentation methods are suitable for use. Besides these requirements, descriptions and analyses are not necessarily tied to specific measurement and segmentation approaches.

Common kinematic descriptions of UE functional motion include plots of kinematics for a single point (12, 13, 26, 97, 127), e.g., position trajectory and velocity magnitude of the wrist. More advanced visualizations include plotting joint angle time series during functional movements (17–21), highlighting compensatory UE motions (136), and visualizations of UE function and activity in free-living environments (103). A comprehensive review of functional UE motion descriptions and analyses is beyond the scope of this paper, with multiple reviews and studies of kinematic analyses already published for UE movements after stroke (16), UE functional impairment measures (8), handwriting (42, 95), and quantifying laparascopic surgical skill (51, 52, 107). Instead, we note a few directions where computer-assisted methods could support the description and analysis phase.

### 5.1. Automating existing measures vs. creating new ones

Given that many existing clinical measures are already validated and well-known by rehabilitation professionals (41), automating these measures may offer additional benefit because clinicians are already familiar with them and would benefit from potential resource or time savings. For example, (137) used machine learning to infer clinically validated scores of UE motor impairment and movement quality in stroke and traumatic brain injury survivors using wearable sensor data. Barth et al. (138) evaluated a method for predicting the UE functional capacity, as defined by the Action Research Arm Test score, of individuals with first-ever stroke using early clinical measures and participant age.

Development, evaluation, and automation of currently non-validated measures, such as some that use kinematic data, should also continue in parallel to automating the output of validated measures. For instance, clinically relevant gait parameters (e.g., walking speed, cadence) and validated gait measures (e.g., the Gait Deviation Index and the Gross Motor Function Classification System score) have been inferred from 2D keypoint human pose estimates using a single RGB camera (79); a methodology which could apply to UE functional motion. An UE-specific example is the development of a kinematic-based quantitative measure of UE movement quality post-stroke from motions performed during two widely used qualitative assessments, where the quantitative measure was found to be strongly correlated with the qualitative assessment results (83). Similarly, a measure of movement quality from UE kinematics of individuals with chronic stroke symptoms captured during a rehabilitation game was evaluated against established UEFAs (84).

Note that kinematic descriptions and analyses tend to be explainable and expert-derived, e.g., in contrast with representation learning. This is largely due to intervention decisions being the responsibility of a human that must be able to interpret the data. However, this does not preclude the use of methods such as deep learning to help with analysis, as is the case with (79).

### 5.2. Time series data mining

Time series data mining techniques have been successfully used for segmenting motions and analyzing skill in the robotic surgery setting (127, 131, 139–141). Some of these methods have also been used for the analysis phase, such as converting trajectories to string representations (e.g., symbolic aggregate approximations (SAX) (127)) and comparing time series using a method called dynamic time warping (DTW) (131, 139, 140). DTW is useful because it allows the measurement of similarity between two time series with varying speeds. The motivation for these works is that surgical motion classes (e.g., grab needle, pull needle, rotate suture once (141)) and surgical skill levels follow distinctive patterns. While movement segmentation are often a focus of these works, the use of DTW represents a direction where kinematic measures are computed based on comparisons, as opposed to computing a measure from an individual’s kinematics only. For example, (139) computed a score based on how the trajectories of the robotic instrument tips compared to “optimal” trajectories during a simulated surgical task. While it is unclear what an optimal trajectory would be in a clinical setting, the time series data mining techniques these surgical motion segmentation and skill evaluation methods use could be relevant for identifying patterns in functional UE motion on standardized tasks.

### 5.3. Dimensionality reduction

As more measures are developed and validated, it is possible that for a particular functional motion there could be many measures used to describe it (49). Another research direction is to use computational methods for visualizing high dimensional data, such as using t-SNE (142), UMAP (143), or principal component analysis (49, 83). Using dimensionality reduction techniques to project high dimensional data to two or three dimensions for plotting could be useful for seeing how the evaluated individual compares to others.

### 5.4. Validation and standardization

The lack of validated and standardized kinematic-based outcome measures are a substantial barrier to more widespread usage of kinematics by domain experts (16). Although domain-specific researchers are likely better positioned to address this problem, computer-assisted tools that make descriptions and analyses easier to acquire will enable a wider group of domain-specific researchers to develop and evaluate kinematic-based outcome measures.

## 6. Assessment and interpretation

Following the definition in Table 1, this phase involves the assessment and interpretation of kinematic-based outcome measures to inform decisions about training or clinical interventions. At this stage in the workflow depicted in Figure 1, the data have been measured, segmented according to the needs of a particular analysis, and descriptions or analyses have been computed. Staying within this review’s scope of developing computer-assisted tools to better support the kinematics analysis process, two areas are considered: (1) automating the assessment and interpretation of kinematic measures, and (2) making descriptions and analyses from the previous workflow stage available to domain experts for interpretation.

### 6.1. Automating assessment and interpretation

Although adoption of kinematic analyses is currently limited, researchers have recently used machine learning and artificial intelligence to automate aspects of the interpretation and assessment process (41, 42, 52, 94, 95, 107, 144–146). Whereas machine learning in the previous section is used to output outcome measures that a domain expert would interpret as part of their decision-making process, the methods considered here automatically output an assessment (e.g., the presence of a disease) based on input kinematic-based outcome measures (see Section 5). Pereira et al. (144) provides a systematic review of machine learning approaches and data sets for inferring the diagnosis of Parkinson’s Disease using kinematic measurements, among other data sources. Classification models trained on kinematic features have been used to predict the skill level of laparoscopic surgeons (52, 107). Handwriting on consumer tablets has been used for automated diagnosis of dysgraphia (95) and neurological disease (42, 94, 145, 146).

### 6.2. Interfacing with kinematic measures

How domain experts physically interface with kinematic-based outcome measures, either from UEFAs or during free living, has also been studied from the perspective of human-centered design (147, 148). These outcome measures are just one of a variety of inputs domain experts use in their assessments, necessitating consideration of how to integrate these various inputs into a system easily used by domain experts. For example, a 2020 survey on requirements for a post-stroke UE rehabilitation mobile application showed that rehabilitation clinicians in the United States and Ethiopia valued the ability to record video of UE function, automatically update performance measures, graphically display patient performance in a number of factors, and see current quality of life and pain levels, among other desired features (147). Similarly, in (148), rehabilitation clinicians qualitatively evaluated a prototype dashboard that visualized UE movement information in stroke patients. The dashboard was then revised based on their feedback and presented in (148). User studies like these will be essential to successfully integrating kinematics analyses into domain expert workflows.

## 7. Outlook

### 7.1. Measurement

A variety of tools are available for measuring kinematics, from low-cost optical and wearable sensors to high-cost optical motion capture systems. Although not offering the same accuracy as optical motion capture, low-cost sensors and pose estimation algorithms provide an opportunity for wider usage of kinematics for specific applications, primarily for measuring gross movements (71, 83, 84). Integration of these more flexible systems will depend on developing more accurate human pose estimation methods that generalize to populations of interest to domain experts (17), measure relevant quantities for the particular domain, e.g., 3D pose and joint angles (38), and are shown to be reliable, responsive, and valid (9, 86). Wider adoption of low-cost measurement sensors depends on whether the data from these systems combined with specific kinematic UEFAs are demonstrated to be valid and reliable, as is done in (83, 84). Alternatively, kinematic UEFAs that indicate an acceptable measurement error range could help identify what movement measurement approaches to use in practice. Ease-of-use is another barrier to using markerless pose estimation methods more widely. Some works have developed software packages that are more accessible (e.g., two or more smartphones can be used, user-friendly application) outside of the laboratory, while also incorporating methodological modifications to improve kinematic quantities (e.g., body models) (62). Ease-of-use may also be why the movement science community has frequently used the Kinect for markerless motion capture (see section 3.2.2.1), where the Kinect has a built in pose estimation capability accessible via a relatively simple application programming interface (API).

### 7.2. Movement segmentation

A variety of methods exist for movement segmentation, which could help automate the processing of data before analysis and interpretation by a domain expert. Movement segmentation, along with measurement, represent the most costly and burdensome parts of the kinematic analysis workflow for UEFAs that computer-assisted methods could help address. Current segmentation workflows used by researchers will not scale to the volume of data expected as kinematic measurements and analyses become more prevalent outside the laboratory. Automated segmentation approaches, along with improved measurement approaches, will enable more widespread kinematic data capture and processing, especially in unconstrained natural environments, e.g., at home. More accessible kinematic data capture and segmentation would give a wider range of domain experts access to kinematics analyses to support the further validation and standardization (16) of kinematics-based outcome measures and the administration of measures that have been validated.

In addition to the outstanding problems of algorithm generalizability and the general lack of algorithm verification due to difficulties with acquiring labeled data (48), motion hierarchies (see Table 3) tend to be inconsistently defined. Although there appears to be agreement across the computational and health literature that there are at least three levels of motion (2, 102), different names for these levels could be confusing to domain experts and limit their application. Consensus on a functional motion hierarchy amongst computational researchers and domain experts will be necessary for segmentation algorithm development. Standardization of a functional motion hierarchy will help researchers curate more relevant data sets, where those data sets will be essential to further development of segmentation algorithms for evaluation and learning-based algorithms. The lack of relevant, rehabilitation-focused data sets that follow a standardized motion hierarchy needs considerable attention by the research community, where the requirements for those data sets will require expertise from both computational researchers and domain experts.

### 7.3. Description and analysis

The validation of kinematic measures is essential to more widespread usage. However, a valid kinematic measure computed using a specific measurement and segmentation approach may not be valid using another type of measurement and segmentation approach, making it difficult to generalize kinematic measures that have not been validated with a particular set of measurement and segmentation methods (9). Furthermore, we believe that computing the kinematic descriptions and analyses themselves is not a burdensome aspect of the kinematic analysis process if the kinematic data are accurate (section 3) and properly segmented (section 4).

Developing methods to more easily measure and output existing validated clinical measures appears to be a valuable direction to pursue because of familiarity and existing use by clinicians (41). In addition to existing measures, developing methods to better measure kinematics, compute kinematic outcome measures, and validate them should be pursued in parallel. An approach to developing and evaluating new quantitative measures from kinematics is to compare the kinematics-based measure to currently used assessments that have been demonstrated to be valid and reliable (83, 84). In healthcare, improving the quality of outcome measures and making the assessments easier to administer are important for patient outcomes and documentation. Outcomes research is used to understand the effectiveness of health services and interventions, or *outcomes*, necessitating outcome measures that are both valid and reliable (149). Furthermore, the need for repeated assessments to inform interventions throughout the rehabilitation cycle (150) necessitates easily acquired and sensitive movement quality measures (9).

### 7.4. Assessment and interpretation

There is currently no consensus on how domain experts should use kinematic measures (9). Additionally, it is currently not known how computer-aided assessments or diagnosis (42) would best integrate into the kinematic analysis workflow used by a domain expert beyond use as a screening tool because of potential biases in the data (e.g., cultural and impairment variations), small reference data sets, and limited data on whether automated systems actually improve health outcomes (39, 41). Although there has been success in automating aspects of robot-assisted surgical skill assessment and handwriting analysis, it is unlikely that domain experts, especially clinicians, will be replaced with fully autonomous systems responsible for deciding on interventions (42). Instead, a potentially more tractable approach is for computer-assisted methods to be designed to assist domain-experts in making decisions by providing more objective information (138).

Integrating artificial intelligence and autonomous systems (41) into domain expert processes is challenging and raises questions about reliability, trust, generalizability, and how domain experts and individuals can interface with the autonomous system. McDermott et al. (151) provides a framework for interviewing domain experts and establishing requirements that can enable an effective human-autonomy partnership. System-level user requirement studies can also inform the integration process (147, 148). Additionally, cognitive systems engineering research could be an area that provides valuable quantitative evaluations on how computational tools integrate into domain expert workflows, such as the recently proposed joint activity testing framework (152). The need for more user-friendly kinematics measurement, segmentation, and analysis methods, as well as investigating how to integrate kinematic analyses into domain expert workflows (i.e., human factors), underscores the multidisciplinary approach required to meaningfully improve the quality and administration of UEFAs.

## 8. Conclusion

Computer-assisted methods could serve an important role in improving outcomes by making kinematic measurement and analysis for UEFAs more accessible and cost-effective, especially for usage in clinics and one’s natural environment. Markerless optical motion capture and automated segmentation algorithms are recent developments that may alleviate some of the most burdensome aspects of the kinematic analysis workflow. However, additional improvements are still needed, along with studies of validity, reliability, explainability, and generalizability for domain-specific UE applications. Better computer-assisted tools for kinematics analysis may also support the further development and evaluation of kinematics-based outcomes measures by giving domain-experts greater access to kinematics data and analysis tools. Furthermore, how best to incorporate kinematic analyses in domain expert workflows in a way that improves health or job-related outcomes remains an open problem. As evidenced by the wide-ranging reach of this review, interdisciplinary collaboration will be critical to developing computational tools that meaningfully support the kinematic analysis process for evaluating functional UE movement.
